# Indications and Complications of Subperiosteal Implants: Literature Review and Case Series

**DOI:** 10.3390/dj13080337

**Published:** 2025-07-23

**Authors:** Gerardo Pellegrino, Maryia Karaban, Carlo Barausse, Amerigo Giudice, Alessandro Antonelli, Roberto Pistilli, Pietro Felice

**Affiliations:** 1 Department of Biomedical and Neuromotor Sciences, University of Bologna, Via San Vitale 59, 40125 Bologna, Italy; 2 Department of Health Sciences of the ‘Magna Graecia’, University of Catanzaro, 88100 Catanzaro, Italy; 3 Oral and Maxillofacial Unit, San Camillo-Forlanini Hospital, 00152 Rome, Italy

**Keywords:** subperiosteal implants, severe jaw atrophy, digital workflow, narrow crest, implant complications, custom implants, prosthetic rehabilitation

## Abstract

**Background/Objectives:** Severe jawbone atrophy, particularly in elderly or medically compromised patients, presents a significant challenge for conventional implant placement. In cases where bone augmentation is not feasible, alternative techniques—such as short, narrow, tilted, and zygomatic implants—may be indicated for the rehabilitation of the atrophic jaw. Subperiosteal implants have re-emerged as a further viable alternative, especially with recent advancements in digital planning and custom fabrication. This study aims to evaluate the clinical outcomes and complications associated with subperiosteal implants through a literature review and a supporting case series, and to propose clinical preliminary guidelines for their use. **Methods**: Fourteen studies—including case reports, case series, retrospective studies, and systematic reviews—were analyzed to assess the effectiveness and risk profile of subperiosteal implants. Additionally, we present a case series of nine patients with advanced vertical and horizontal alveolar bone atrophy treated using custom-made, digitally-designed subperiosteal implants. Surgical techniques, prosthetic workflows, and complications were recorded and assessed. **Results**: Subperiosteal implants were found to be particularly suitable for patients with narrow alveolar crests and severe atrophy where traditional implants are contraindicated. Literature and case series data indicated favorable outcomes, with early complications such as soft tissue inflammation and prosthetic misfit being manageable. A low complication rate was recorded in our series, with digital workflows contributing to improved implant fit and reduced technical errors. **Conclusions**: Subperiosteal implants could offer an effective solution for complex atrophic cases, provided that patient selection, surgical precision, and prosthetic design are meticulously managed. Based on our findings, clinical recommendations are proposed to guide their application in contemporary practice.

## 1. Introduction

Severe bone atrophy in the alveolar ridge has long posed a significant challenge in dental implantology. This condition often arises following tooth extraction due to periodontal disease, trauma, or congenital defects, leading to progressive bone loss in the maxilla or mandible [1]. As bone volume decreases, achieving fixed rehabilitation with dental implants becomes increasingly difficult, as successful osseointegration requires a stable and sufficiently dense foundation [2]. Patients with significant bone loss frequently experience impaired speech, difficulty chewing, and reduced self-esteem due to the aesthetic and functional consequences of tooth loss, such as sunken cheeks and a decreased vertical dimension of the lower face [3,4].

To address these challenges, various surgical techniques have been developed. These include sinus lifting and zygomatic implant placement in the maxilla, as well as inlay and onlay bone grafting, guided bone regeneration (GBR) followed by standard implant placement, and the use of tilted, narrow, and short implants in both arches [5,6,7,8,9,10,11,12,13,14,15,16]. While these surgical interventions can improve function, aesthetics, and overall quality of life, they present certain limitations. They often require multiple surgical stages, involve extended healing periods, increase treatment costs, and do not always allow for immediate prosthetic rehabilitation. Additionally, failure of osseointegration can result in implant or prosthetic failure, further complicating the clinical outcome [17,18,19,20,21,22,23].

Recent technological advancements—including digital imaging, 3D printing, and customized implant design—have significantly improved treatment options for patients with severe bone deficiencies. One notable development is the renewed interest in subperiosteal implants, which offer a viable alternative for patients who are not suitable candidates for traditional endosseous implants [24,25,26,27]. These implants, placed on the bone surface rather than within it, present a promising solution when bone grafting or osseointegrated intraosseous implants are not feasible. Recent studies have focused on enhancing the precision, survival rates, and patient satisfaction associated with subperiosteal implants, marking a shift in treatment strategies for complex cases of bone atrophy [14,15,16]. Unfortunately, despite these advancements, there are still no clearly defined clinical guidelines for the use of subperiosteal implants, and their indications, surgical protocols, and long-term outcomes remain insufficiently standardized [28,29,30,31,32,33].

As research and technology continue to evolve, implant dentistry is expanding the range of treatment possibilities for patients with severe bone loss [34]. This study aims to provide a comprehensive literature review on subperiosteal implants, examining current applications, clinical outcomes, and recent advancements in technology and design for managing severe bone atrophy in implant dentistry. In addition, a case series based on the authors’ clinical experience is presented to contribute practical insights and propose preliminary guidelines for the effective application of subperiosteal implants in modern clinical practice.

## 2. Materials and Methods

### 2.1. Literature Review

This systematic literature review follows PRISMA (Preferred Reporting Items for Systematic Reviews and Meta-Analyses) guidelines to ensure transparency and reproducibility. The study protocol was designed to minimize selection bias and ensure a comprehensive evaluation of the available literature [35].

### 2.2. Inclusion Criteria

- Human clinical studies, including clinical trials, cohort studies, case-control studies, case series, case reports, review articles, letters, editorials, expert opinions, and systematic reviews.
- Studies evaluating survival rates, osseointegration, or complications of subperiosteal implants.
- Studies comparing subperiosteal implants with conventional implants or alternative treatment modalities.
- English-language publications to ensure accessibility and consistency in interpretation.
- No time restrictions to include both historical and modern studies, allowing for a comprehensive perspective on subperiosteal implants’ evolution and clinical outcomes.

### 2.3. Exclusion Criteria

- Animal or in vitro studies that lack direct clinical implications.
- Studies focusing solely on technical or laboratory procedures without reporting clinical outcomes.

### 2.4. Search Strategies and Information Sources

A comprehensive literature search was conducted across the following electronic databases: PubMed, Scopus. The search strategy incorporated controlled vocabulary (MeSH terms) and free-text keywords to maximize sensitivity. The search was performed using the following Boolean operators:

((“subperiosteal implants” OR “subperiosteal dental implants” OR “bone-implant interface” OR “custom implants” OR “periosteal implants”) AND (“implant survival” OR “implant failure” OR “long-term success” OR “complications” OR “implant durability” OR “implant prognosis” OR “implant complications” OR “prosthetic failure” OR “implant failure prognosis”)) AND “humans”

Manual screening of reference lists from selected studies was also conducted to capture additional relevant publications.

### 2.5. Article Selection

The article selection process was conducted in multiple stages:

1. Primary Search Results:

Two independent reviewers (M.K. and S.T.) screened search results from PubMed (264 articles), Scopus (452 articles).

2. Duplicate Removal:

Duplicate articles were identified and removed using Rayyan System (Rayyan Systems Inc.), resulting in the exclusion of 476 duplicate records.

3. Title and Abstract Screening:

Titles and abstracts were screened for relevance using Rayyan software. Each reviewer categorized articles as “include,” “exclude,” or “undecided.” A third reviewer (C.B.) acted as an arbitrator to resolve discrepancies and reviewed articles marked as “undecided.” After this screening process, 23 articles were selected for full-text evaluation.

**Figure 1:**
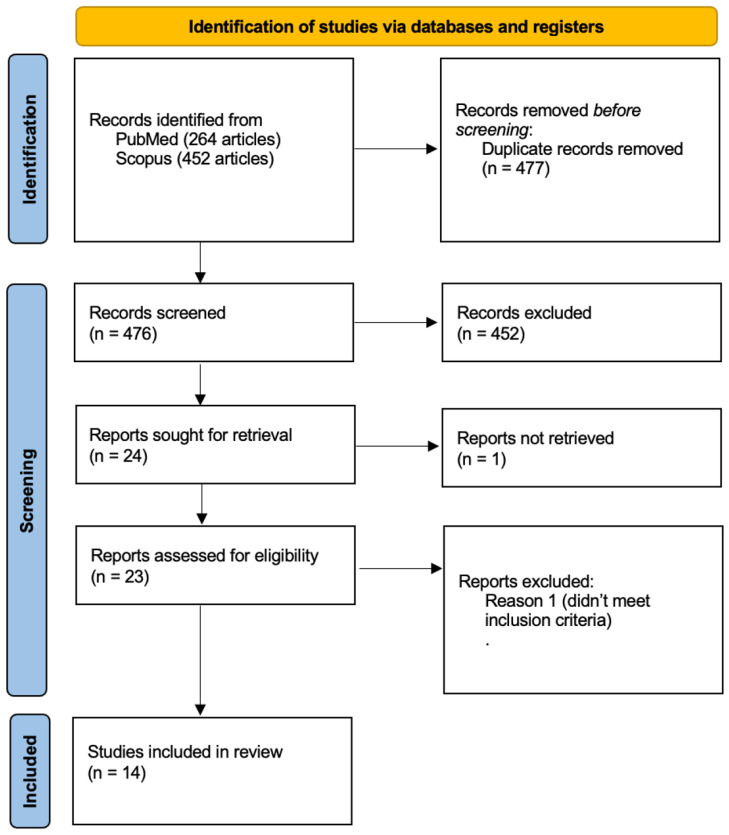
The flowchart visually represents the systematic selection process for studies included in the review, detailing identification, screening, eligibility, and final inclusion criteria. Fourteen studies met all requirements and were included in the final review.

4. Full-Text Evaluation:

Full texts of the selected articles were assessed for adherence to eligibility criteria and methodological quality. Studies that lacked sufficient clinical data or failed to report survival rates, complications, or patient follow-up information were excluded. The final selection included studies that provided relevant and high-quality clinical evidence on subperiosteal implants. Fourteen articles were selected for data extraction (Figure 1) [36,37,38,39,40,41,42,43,44,45,46,47,48,49].

### 2.6. Data Extraction Process

Data extraction was performed systematically to ensure consistency and accuracy. All information from the selected articles was extracted into an Excel spreadsheet for structured analysis. The following variables were collected from each included study:

- Study Characteristics: Author(s), year of publication, study title, journal name, and DOI/link to ensure accurate identification and citation of the sources.
- Study Design and Population: Study design, sample size, and study location/country to assess the methodological quality and generalizability of findings.
- Follow-up and Patient Characteristics: Follow-up period, patient demographics, jaw treated (maxilla, mandible, or both), and bone condition to analyze treatment outcomes in different clinical scenarios.
- Implant Characteristics: Type of implant material and fixation method to compare variations in design and placement techniques.
- Outcome Measures: Reported survival rates and success criteria used in each study to evaluate implant longevity and clinical performance.
- Complications and Prosthetic Considerations: Documented complications, prosthetic type, and patient satisfaction levels to assess functional and patient-reported outcomes.
- Key Findings and Study Limitations: Major conclusions, clinical implications, and limitations noted by the authors to contextualize study results.

Extracted data were systematically categorized and analyzed to identify trends, patterns, and potential correlations among study variables. Quantitative outcomes, such as survival rates and complication frequencies, were synthesized descriptively (Table 1).

### 2.7. Case Series

This retrospective case series includes nine patients with 11 sites who underwent jaw rehabilitation with 11 custom-made subperiosteal implants between 2020 and 2024 at the Department of Oral Surgery, University of Bologna. All patients presented with severe alveolar ridge atrophy and were deemed unsuitable for conventional endosteal implants due to insufficient bone volume or anatomical limitations. Written informed consent was obtained from all patients.

### 2.8. Patient Selection and Preoperative Assessment

Inclusion criteria consisted of:

- Severe mandibular or maxillary bone atrophy.
- Contraindication to bone grafting or patient refusal of grafting procedures.
- Need for fixed implant-supported rehabilitation.
- Good general health and absence of uncontrolled systemic conditions.

Exclusion criteria consisted of:

- Presence of active oral infections or untreated periodontal disease.
- Uncontrolled systemic conditions (e.g., diabetes, cardiovascular disease, immunosuppressive disorders) or history of head and neck radiation therapy.
- Severe parafunctional habits (e.g., bruxism) that could compromise implant stability.
- Pregnancy or breastfeeding at the time of treatment.

All patients underwent a comprehensive clinical evaluation, including panoramic radiographs and Cone Beam Computed Tomography (CBCT). Digital intraoral scans or conventional impressions were used to assess soft tissue contours and plan implant design. Particular attention was given to the evaluation of mandibular anatomy, mental foramen position, and residual bone morphology.

Custom subperiosteal implants were designed using patient-specific CBCT and intraoral scans. Implant design aimed to achieve passive adaptation to the cortical surface of the mandible and to avoid impingement on critical anatomical structures. Prosthetic abutment positioning was virtually planned based on prosthetic requirements and opposing arch relationships. All implants were manufactured in titanium alloy using selective laser melting technology (BTK, Biotek SRL) and delivered with a custom-made surgical guide.

Surgical procedures were performed under local anesthesia (Articaine 4% with Epinephrine 1:100,000). A mid-crestal incision was made along the edentulous ridge with posterior releasing incisions to allow full-thickness mucoperiosteal flap elevation. The mental nerves in the mandible were carefully identified and protected throughout the procedure. Minimal osteoplasty was performed in areas of bone irregularities to ensure a passive fit of the implant framework. Using the predesigned surgical guide, the implant was positioned and fixated with titanium screws at predetermined anchorage points. The number and position of fixation screws were adapted to each patient’s anatomy to ensure primary mechanical stability.

Soft tissue closure was achieved with resorbable sutures (Vicryl 4-0), and a panoramic radiograph was obtained immediately postoperatively to verify implant positioning. Provisional prostheses were delivered within 24–72 h post-surgery to preserve occlusal function and assist soft tissue contouring. Materials included milled PMMA or reinforced resin frameworks depending on case needs. Definitive prostheses (zirconia or metal-ceramic) were fabricated after a healing period of 4 months, following confirmation of soft tissue stability and implant integrity. All patients received antibiotic prophylaxis (Amoxicillin 2 g preoperatively, followed by 1 g twice daily for 7 days), anti-inflammatory agents (Ibuprofen 600 mg as needed), and Chlorhexidine 0.20% mouthwash twice daily for 1–2 weeks. Sutures were removed at 10–14 days.

Patients were followed up at 1, 3, and 6 months postoperatively, and every 6–12 months thereafter. Follow-up assessments included clinical examinations, panoramic radiographs, CBCT (when indicated), and prosthetic evaluations. Maintenance hygiene protocols included semiannual professional cleaning and individualized oral hygiene instructions.

## 3. Results

### 3.1. Literature Review

A total of 14 studies were included in the analysis. The included studies employed diverse methodologies. Retrospective clinical studies were conducted by Cerea & Dolcini (2018), Onică et al. (2024), Ayhan et al. (2024), Vaira et al. (2024), and systematic reviews were performed by Anitua et al. (2024) and El-Sawy & Hegazy (2024). Case series included Mangano et al. (2020), Gellrich et al. (2024), and Santiago et al. (2025), while pilot studies and single-case reports were documented by Nemtoi et al. (2022), Marconcini et al. (2023), Strappa et al. (2022), and Ayhan et al. (2024) [36,37,38,39,40,41,42,43,45,47,49]. A comparative clinical study was performed by Zielinski et al. (2025) [48]. The largest retrospective study by Anitua et al. (2024) analyzed 227 implants, while smaller case series and single-case reports covered samples ranging from 1 to 36 patients [46]. The cumulative sample consisted of 617 patients with severe maxillary or mandibular atrophy, often classified as Cawood and Howell Class V–VI. Most patients were elderly (mean age range: 60.4–69.6 years), although one case involved a young patient with ectodermal dysplasia [50].

### 3.2. Patient Demographics and Clinical Indications

Patients were predominantly edentulous with severe jaw atrophy, often classified as Cawood and Howell Class V or VI. The average age was typically above 60, with exceptions such as the 18-year-old patient with ectodermal dysplasia reported by Ayhan et al. (2024) [42]. Several studies included medically compromised patients, such as those with osteoporosis (Marconcini et al., 2023) or generalized systemic conditions (El-Sawy & Hegazy, 2024) [40,47].

### 3.3. Follow-Up Periods and Duration of Evaluation

Follow-up durations ranged from 6 months (Santiago et al., 2025) to up to 6 years (Onică et al., 2024), although the latter reported a low long-term survival rate (~25%). Most studies had follow-up periods of 12–24 months [41,49]. Gellrich et al. (2024) included follow-up up to 68 months, and Zielinski et al. (2025) evaluated outcomes after a minimum of 5 years, highlighting the long-term feasibility of subperiosteal implants [43,48].

### 3.4. Survival Rates and Success Criteria

Implant survival rates were generally high, ranging from 86.7% (Ayhan et al., 2024) to 100% in several studies (Mangano et al., 2020; Strappa et al., 2022; Gellrich et al., 2024) [37,38,42,43]. The lowest survival (around 25%) was reported by Onică et al. (2024) over a long-term 6-year period [41]. Success was commonly defined by implant stability, absence of complications, and functional prosthetic integration.

### 3.5. Complications and Clinical Outcomes

Complications emerged as a central concern in the evaluation of subperiosteal implants, despite their generally high survival rates. While most studies reported favorable outcomes, the risk of both early and late complications was consistently present.

### 3.6. Postoperative and Early Complications

Mild postoperative issues, such as pain, swelling, and soft tissue inflammation, were commonly observed. Mangano et al. (2020), Vaira et al. (2024), and Santiago et al. (2025) noted transient edema and discomfort, which typically resolved within days [37,45,49]. However, more severe early complications were reported. Nemtoi et al. (2022) described early implant failure due to postoperative infection, while Ayhan et al. (2024) reported a 13.3% implant failure rate, primarily due to infection and inadequate osseointegration. Onică et al. (2024) provided a particularly critical perspective, with 17 out of 67 implants showing mobility over a 6-year follow-up, suggesting potential design flaws or long-term biological complications [39,41,42].

### 3.7. Prosthetic Complications

Prosthetic challenges were also noted in several studies. Mangano et al. (2020) reported fractures of provisional restorations in two cases, although these did not compromise the final outcome [37]. Ayhan et al. (2024) encountered significant prosthetic misfit requiring intraoperative adjustments, indicating that manufacturing precision remains a clinical concern even with digital workflows [42]. Additionally, mucosal dehiscence requiring surgical intervention was documented in Strappa et al. (2022) [38].

### 3.8. Long-Term and Late Complications

Long-term complications, though less frequently reported, raised important questions regarding the stability and integration of subperiosteal implants over time. Zielinski et al. (2025), in a comparative study with 5 years of follow-up, observed mucosal inflammation and prosthesis-related complications but reported no implant failures. On the other hand, the high rate of late implant mobility described by Onică et al. (2024) (25% survival at 6 years) reflects the potential for mechanical or biological degradation over time [41,48].

### 3.9. Infection and Implant Loss

Infection was one of the most commonly cited serious complications. In addition to the early infections seen in Nemtoi et al. (2022) and Ayhan et al. (2024), Marconcini et al. (2023) noted marginal bone exposure and inflammation, though without implant loss. These findings emphasize the importance of patient selection, surgical technique, and meticulous postoperative care in minimizing infectious complications [39,40,42].

### 3.10. Summary of Risk Profile

Studies with longer observation periods—such as Onică et al. (2024) and Zielinski et al. (2025)—tended to report more complications, highlighting the importance of long-term monitoring [41,48].

### 3.11. Case Series

#### 3.11.1. Patient Demographics and Clinical Characteristics

The case series included nine patients (five females and four males), aged between 27 and 69 years, all of whom presented with vertical bone atrophy of the maxilla or mandible that precluded the placement of conventional endosteal implants. One patient had a syndromic condition—EEC syndrome (ectrodactyly–ectodermal dysplasia–cleft syndrome)—while no patients presented systemic contraindications to surgery.

#### 3.11.2. Surgical and Prosthetic Protocol

**Figure 2:**
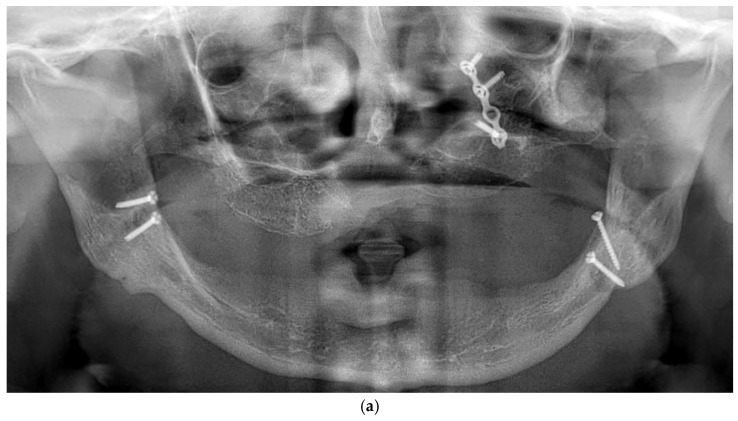

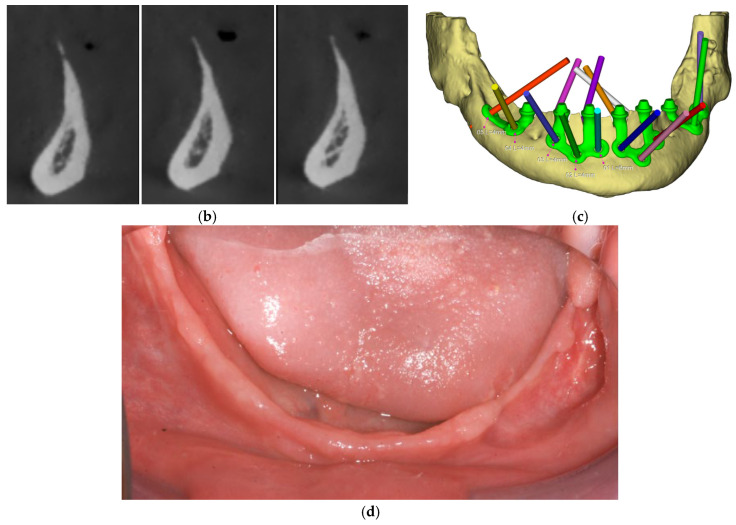
(**a**) A panoramic radiograph of the patient at the initial stage was obtained to evaluate the overall dentition, bone structure, and any developmental anomalies. (**b**) Cross-sectional images show a severely resorbed, knife-edged alveolar bone crest. (**c**) Digital planning of the subperiosteal implant design and screw positioning. (**d**) Intraoral clinical image showing a completely edentulous knife-edged alveolar ridge.

All patients were rehabilitated using custom-made subperiosteal implants, designed from the patients’ CBCT DICOM files to precisely match the anatomical features of each jaw. A fully digital workflow was used in all cases, including the RealGuide^®^ software 5.4 for planning. The implants were designed as segmented frameworks, with two to four divisions per arch and 2–3 abutments per segment (Figure 2a–d).

**Figure 3:**
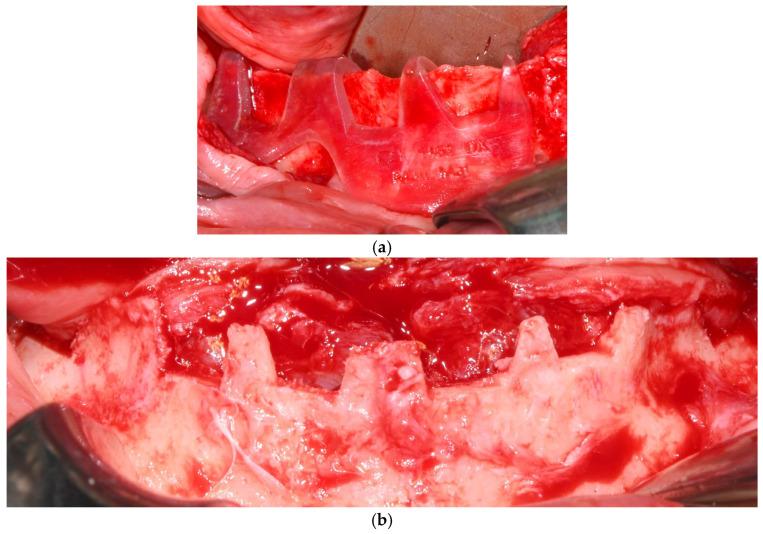
(**a**) Surgical guide indicating the osteoplasty to be performed on the left side of the mandible. (**b**) Post-osteoplasty view showing the entire mandibular crest.

Surgical procedures followed a standardized protocol. A mid-crestal incision was made along the edentulous ridge with releasing incisions as needed, followed by full-thickness mucoperiosteal flap elevation to expose the underlying bone. All the nervous structures were carefully identified and isolated to avoid iatrogenic damage. Reference points for implant adaptation were confirmed using a pre-designed surgical guide. In some cases, minor osteoplasty using a surgical guide was performed to regularize bone contours and enhance implant adaptation (Figure 3).

**Figure 4:**
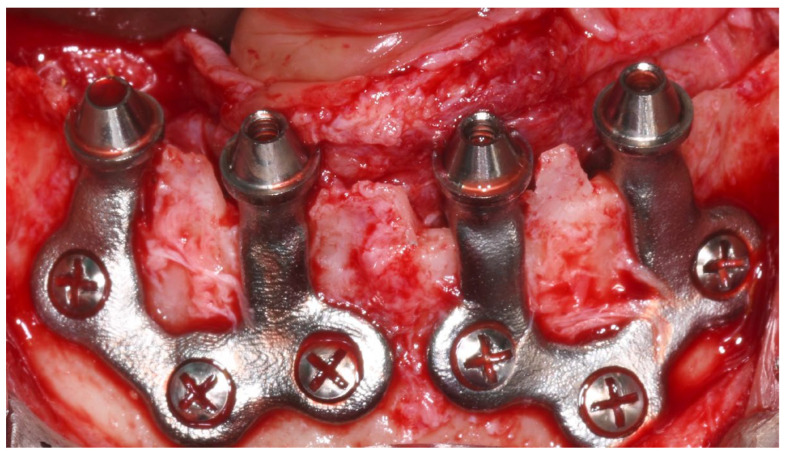
Intraoperative view of the mandibular crest showing the placement of subperiosteal implants during surgery.

Each subperiosteal implant was passively adapted to the bone and secured with titanium osteosynthesis screws to achieve primary stability (Figure 4).

**Figure 5:**
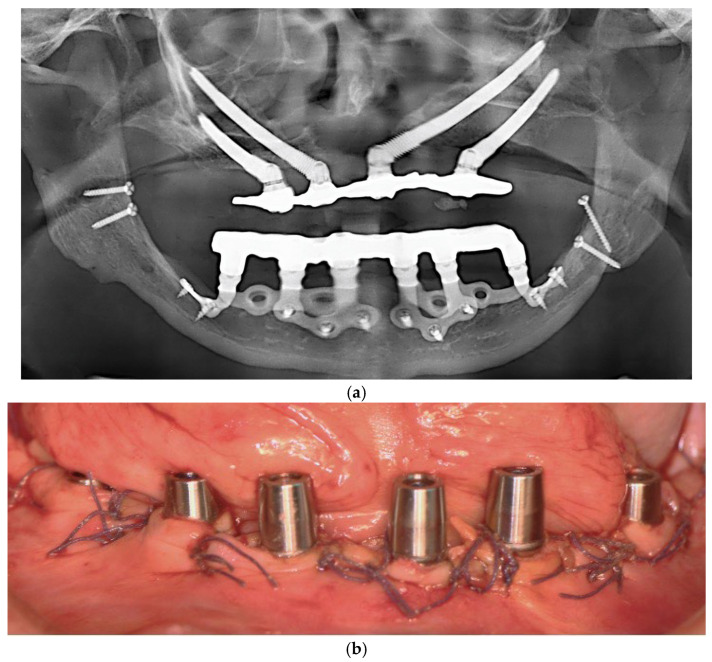
(**a**) A panoramic radiograph of the patient after subperiosteal implants placement. (**b**) Intraoral picture of the mandible following subperiosteal implant placement.

Flaps were repositioned and sutured with resorbable Vicryl 4-0 sutures. Panoramic radiography and clinical pictures were obtained postoperatively (Figure 5a,b). Within 48 h, digital impressions were taken, and a provisional screwed PMMA prosthesis was delivered. In all patients, immediate loading was achieved without intraoperative complications. Postoperative care included Amoxicillin 1 g twice daily for 7 days, Ibuprofen 600 mg as needed, and Chlorhexidine 0.20% mouthwash twice daily for 14 days. Sutures were removed after 14 days.

Final prosthetic delivery occurred approximately 3 months post-surgery, following confirmation of soft tissue healing and implant stability. Prosthetic materials included PMMA on a metal bar or zirconia frameworks.

**Figure 6:**
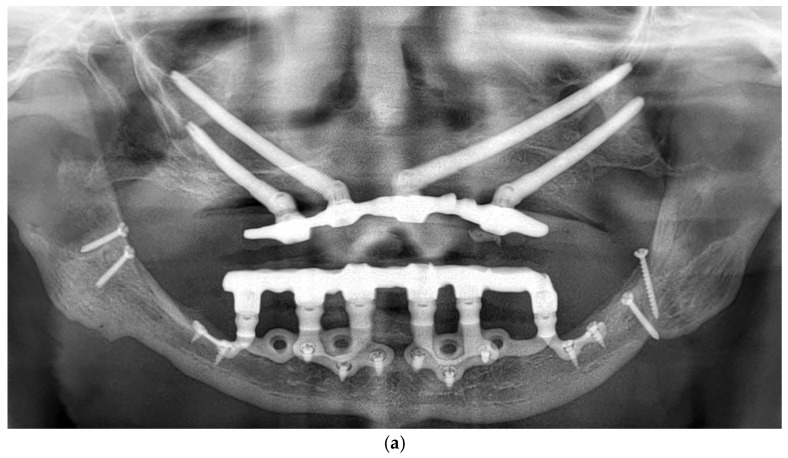

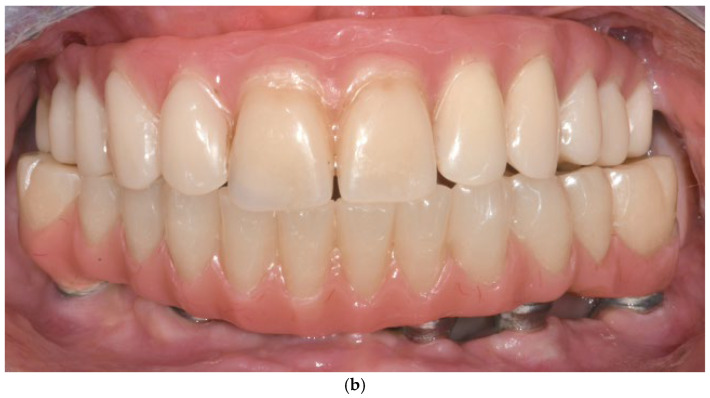
(**a**) A panoramic radiograph of the patient at 5-year follow-up. (**b**) Intraoral clinical image of the patient at 5-year follow-up.

Patients were followed up at 3 and 6 months postoperatively, and then annually. Follow-up assessments included clinical evaluation and panoramic imaging; CBCT scans were obtained when necessary to monitor implant fit, peri-implant tissue response, and bone contour stability. All implants remained functionally stable throughout the observation period. The follow-up period ranged from 12 to 60 months, with a mean duration of 36.2 months (Figure 6a,b). Each patient underwent routine hygiene sessions every six months and an annual special session with prosthesis removal for thorough cleaning.

#### 3.11.3. Complications and Management

A total of three minor complications were observed, corresponding to a complication rate of 37.5%. All complications were managed conservatively and did not require surgical re-intervention (Table 2).

- Patient 1: Developed a localized abscess in the region of tooth 13 three months postoperatively, accompanied by soft tissue dehiscence at sites 13, 15, and 17. These were managed with local irrigation and hygiene reinforcement, with full recovery.
- Patient 2: Presented with tissue dehiscence on the lingual side.
- Patient 3: Presented with tissue dehiscence around the second abutment of the upper left implant. The condition was resolved through improved oral hygiene.

There were no reports of early implant failure, screw loosening, prosthetic fracture, or exposure of the implant framework in any case.

## 4. Discussion

The review of 14 studies investigating the use of subperiosteal implants in patients with severe jawbone atrophy reveals a mixture of positive outcomes and notable complications, which are important for assessing the viability of this treatment option. The studies included in this review span a wide range of methodologies, sample sizes, and follow-up periods, contributing to a comprehensive understanding of the effectiveness and challenges associated with these implants.

The patient populations in the reviewed studies generally consisted of elderly individuals, typically over 60 years old, which corresponds to the progressive nature of jawbone atrophy. This finding is in line with the studies by Anitua et al. and El-Sawy et al., where subperiosteal implants were primarily used in older adults for whom conventional implant placement was not feasible due to anatomical limitations [46,47]. However, certain studies, such as Ayhan et al. (2024), included younger individuals with congenital or syndromic conditions like ectodermal dysplasia, demonstrating the versatility of subperiosteal implants in managing both age-related and developmental bone deficiencies [42]. Cerea et al. (2019) further broadened this scope by treating patients who had previously failed conventional implant therapy, underscoring subperiosteal implants as a last-resort solution in complex cases [36].

Follow-up durations varied significantly across studies, ranging from 6 months to 6 years. Longer follow-up durations, such as those reported by Zielinski et al. (2025) and Onică et al. (2024), showed a higher incidence of late complications, suggesting potential challenges in long-term maintenance [41,48]. In contrast, Anitua et al. reported stable outcomes over a 17-month period with no implant failures, and El-Sawy et al. reported similar success over an average of 18 months [46,47]. Cerea et al., with up to 36 months of follow-up, observed a few soft tissue complications, confirming the need for extended monitoring to fully assess durability [36]. These differences indicate that while short- and medium-term results are promising, long-term data are necessary for a conclusive evaluation.

Complications ranged from mild and self-limiting to more serious issues affecting treatment success. Early postoperative complications—pain, swelling, and inflammation—were common but typically manageable, as confirmed by the experiences in Anitua and El-Sawy ‘s studies [47]. More severe issues such as infection and implant failure were less frequent but notable. Ayhan et al. (2024) reported a 13.3% failure rate primarily due to infection, while Nemtoi et al. (2022) also linked failure to infection [39,46]. Cerea et al. (2019) documented one case of implant loss due to infection in a smoker, highlighting the importance of patient-related risk factors [36]. These findings suggest that success is highly dependent on surgical technique, systemic health, and postoperative care.

Prosthetic challenges such as misfit and provisional restoration fractures were reported in several studies. The adoption of fully digital workflows by Anitua, El-Sawy, and Ayhan was associated with reduced prosthetic complications [42,46,47]. These workflows enable high-precision implant manufacturing and better adaptation to patient anatomy, which contributes to improved outcomes and patient satisfaction. However, even with digital advancements, Cerea et al. noted occasional prosthetic issues, especially in early designs, emphasizing that digital tools, while beneficial, are not foolproof [36]. The most frequent complication remains implant exposure. Its impact on the long-term survival rate of these implants, as well as its association with the onset of mucositis, remains unclear.

Infection remains one of the most concerning complications due to its impact on implant survival. Nemtoi et al. (2022), Ayhan et al. (2024), and Marconcini et al. (2023) all reported infection as a leading cause of early implant loss [39,40,44]. These contrasting results highlight the critical role of surgical protocols and the potential benefits of minimally invasive techniques in reducing infection risk.

Several implant designs and manufacturing techniques were used by different authors, along with varying surgical approaches. These factors further increase the uneven nature of the available literature. However, more clinical data and longer follow-up periods are needed to better evaluate clinical outcomes and establish definitive guidelines [34]. In vitro data based on finite element analysis may be useful for understanding the biomechanical properties of subperiosteal implants, particularly when conducted using realistic virtual models.

In our case series of nine patients treated with custom-made subperiosteal implants using a fully digital workflow, clinical outcomes were favorable. The overall complication rate was low: only three minor cases of localized soft tissue dehiscence occurred, all of which were resolved with conservative measures. No instances of early implant failure, screw loosening, or prosthetic fractures were observed. These findings align with existing literature and support the potential effectiveness of subperiosteal implants in the rehabilitation of severely atrophic ridges, particularly when enhanced by modern digital planning and manufacturing protocols. Notably, the surgical procedure is less invasive and can typically be performed under local anesthesia, with postoperative complications manageable in an outpatient setting. This contrasts with alternative solutions like zygomatic implants, which often require general anesthesia and carry greater surgical risks.

Across the reviewed studies, fixed full-arch prosthetic solutions were the preferred choice. Cerea and Dolcini (2019), Mangano et al. (2022), Marconcini et al. (2023), Nemtoi et al. (2022), and Onică et al. (2024) all reported using fixed restorations—either screw-retained or cemented—with definitive materials such as ceramic or zirconia [36,37,39,41]. Strappa et al. (2022) used a cement-retained Toronto Bridge [38]. Ayhan and colleagues, in multiple studies (2023; 2024), also employed full-arch fixed prostheses, including cases of immediate loading [42,46]. In this case series, the authors exclusively adopted screw-retained full-arch prostheses supported by custom subperiosteal frameworks. This decision was based on their retrievability, structural stability, and ease of hygiene maintenance. No mechanical or prosthetic complications occurred during the observation period, and patient satisfaction was high.

When compared with other implant strategies, subperiosteal implants present a distinct set of advantages and limitations. Zygomatic implants provide stable anchorage in cases of advanced maxillary atrophy but are associated with higher morbidity and potential complications such as sinusitis or oroantral communication. Short and tilted implants offer a less invasive alternative but may lack long-term biomechanical stability in severely resorbed jaws. Subperiosteal implants, by contrast, are custom-designed to conform to the bone surface, allowing for stable prosthetic support even in cases of extreme bone loss—especially when vertical height must be preserved. However, their success depends on adequate soft tissue coverage, and complications such as exposure or prosthetic misfit can still occur, despite the precision offered by digital workflows. Based on the results of our case series and supported by the reviewed literature, we propose the following clinical preliminary guidelines for the successful application of subperiosteal implants.

### 4.1. Patient Selection

- Patients with severe alveolar bone atrophy (Cawood and Howell Class V–VI). (Ideal for those with advanced horizontal and vertical bone loss where standard implants are contraindicated).
- Narrow alveolar crest cases unsuitable for endosseous implants. (Allows rehabilitation without vertical bone reduction. Subperiosteal implant is the only technique that allows for the rehabilitation of narrow bone crests without the loss of vertical height).
- Elderly or systemically stable patients unwilling or unable to undergo bone grafting. (Minimally invasive alternative to augmentative procedures) [50].

### 4.2. Digital Planning

- Fully digital workflows.
- High-resolution CBCT scan without artifacts. (Provides accurate anatomical data for implant customization).
- Use of prosthetically driven planning software. (Ensures correct abutment angulation and emergence profile).
- Collaborative workflow between surgeon, prosthodontist, and technician. (Aligns surgical feasibility with prosthetic functionality) [26].

### 4.3. Surgical Technique

- Full-thickness flap elevation with identification of vital structures. (Prevents nerve injury and ensures proper implant bed access).
- Minimal osteoplasty when needed for passive fit. (Improves implant adaptation to the bony surface).
- Fixation with titanium screws at preplanned anchorage points. (Provides mechanical stability without intraosseous engagement).
- Strict aseptic handling of the implant. (Reduces risk of postoperative contamination).

### 4.4. Implant Manufacturing and Fitting

- High-precision manufacturing and appropriate surface treatments are essential to allow a successful rehabilitation.
- The implant must achieve a passive sitting and fixation, avoiding biomechanical stress on both the structure and fixation screws, as well as the aseptic implant handling [51].

### 4.5. Prosthetic Design

- Immediate loading with provisional prosthesis (24–72 h post-op). (Supports soft tissue healing and functional rehabilitation).
- Final prosthesis placement after 4 months. (Ensures tissue maturation and long-term durability).
- Passive fit and absence of considerable cantilevers. (Prevents biomechanical overload and prosthetic complications) [52].

### 4.6. Postoperative Protocols and Long-Term Monitoring

- Scheduled evaluations at 1, 3, and 6 months post-surgery, then annually. (Allows early detection of complications and implant integration monitoring).
- Annual prosthesis removal and peri-implant hygiene assessment. (Enables thorough decontamination and inspection of framework integrity).
- Customized hygiene instructions and use of chlorhexidine rinses. (Minimizes plaque accumulation and soft tissue inflammation) [53,54].

This study has several limitations that should be acknowledged. First, the retrospective nature of our case series introduces inherent selection bias. The small sample size (n = 9) limits statistical power and generalizability. Follow-up durations varied across cases and were limited in some instances, which may not capture long-term outcomes such as framework degradation or chronic soft tissue changes. Additionally, the absence of a control group treated with alternative methods (e.g., zygomatic or short implants) precludes direct comparative analysis. Despite these limitations, our findings provide valuable preliminary insights and support further prospective, controlled studies with larger patient cohorts.

## 5. Conclusions

Subperiosteal implants represent a promising alternative for the rehabilitation of patients with advanced jawbone atrophy. While short to medium-term outcomes are encouraging—particularly with the integration of digital technologies. The long-term clinical data and standardized protocols are still essential to optimize success and minimize complications. Currently, a narrow alveolar crest is considered the most suitable clinical indication for subperiosteal implants, as no other technique offers a comparable ability to preserve vertical bone height. Implant exposure remains one of the most unwanted complications; however, its impact on long-term success rates is still unclear.

## Figures and Tables

**Table 1:** Main characteristics of the studies included in the review. N/A—not applicable; N/R—not reported; DLMS—direct laser metal sintering.

Author(s), Year	Study Design	Sample	Average Age	Population	Systemic Conditions	Follow-Up	Implant Type/ Implant Brand	Survival	Success Criteria	Complications
Cerea & Dolcini, 2018 [36]	Retrospective	70	67.8	Elderly with jaw atrophy	Excluded smokers and bruxists	2 years	Custom-made DMLS titanium; surface not specified; (Eagle-Grid, BTK, Dueville, Vicenza); polished surface	95.8%	Implant and restoration function	5.7% postop symptoms; 1.4% infection; 8.9% prosthetic issues
Mangano et al., 2020 [37]	Case Series	10	69.6	Elderly, mandibular atrophy	N/R	1 year	Custom 3D-printed titanium (DMLS); (Iuxta3D ^®^, BTK, Dueville, Vicenza, Italy); porous surface	100%	Stable fit, no infection	10% discomfort; 20% provisional fractures
Strappa et al., 2022 [38]	Case Report	1	67	67 y/o female, maxillary atrophy	None	2 years	DMLS titanium alloy; (Eagle-Grid, Eagle-Grid S.r.l., Bergamo, Italia)	100%	No complications	None
Nemtoi et al., 2022 [39]	Pilot Study	16	N/R	Severe resorption	2 diabetes; 1 cardiovascular case	Several months	DMLS titanium (CBCT-designed); (3D Medica SABETTIMED^®^ and Bone Easy^®^; polished and rough surface	93.75%	Stability, integration	1 failure due to infection
Marconcini et al., 2023 [40]	Case Report	1	72	Elderly, osteoporosis	Osteoporosis	1 year	3D-printed titanium; (3Dfast srl, Padova (Italy); porous surface	100%	Implant stability	None
Onică et al., 2024 [41]	Retrospective	36	59.7	Edentulous, severe atrophy	NR	6 years	CAD/CAM titanium; (Sisma S.p.A., Piovene Rocchette, Italy)	~25%	Long-term function without complications	Early exposure, mobility in 27/36 cases
Ayhan et al., 2024 [42]	Case Report	1	18	18 y/o, ectodermal dysplasia	Ectodermal dysplasia	N/A	Custom DMLS titanium; polished and rough surface	N/A	Oral function restoration	None reported
Gellrich et al., 2024 [43]	Case Series	4	N/R	Severe bone loss	Not specified	Up to 68 mo	Patient-specific titanium framework; IPS Implants. Preprosthetic (KLS Martin Group, Tuttlingen, Germany); polished surface	100%	Stability maintained	None
Ayhan et al., 2024 [44]	Retrospective	31	N/R	Severe bone loss	NR	15 months	3D-printed titanium; NR	86.7%	Function, adaptation	Fit issues (23); soft tissue (12); infections (5)
Vaira et al., 2024 [45]	Retrospective	17	61.5	Posterior mandible atrophy	N/R	7–53 mo	DMLS double-laser titanium; (B&B Dental, San Pietro in Casale, Italy); porous surface	100%	Stable implants	Hypoesthesia (transient); edema
Anitua et al., 2024 [46]	Systematic Review	227	N/R	Bone atrophy	Included diabetes, cardiovascular, smoking	21.4 mo	Various, mainly titanium; N/A	97.8%	Functionality maintained	25.6% exposure; 7.5% postop issues
El-Sawy & Hegazy, 2024 [47]	Systematic Review	302	N/R	Atrophic jaws incl. med. compromised	Included diabetes, hypertension, cancer	17.2 mo	Titanium/PEEK blends in some studies; N/A	95.3%	Functional with minor issues	11.5% bio issues; 5.9% prosthetic problems
Zielinski et al., 2025 [48]	Comparative Study	150	N/R	Maxillary atrophy	Included smokers and immunocompromised	≥5 years	CAD titanium; roughened surface in select designs; Mai Implant^®^ (Integra Implants^®^, Lodz, Poland)	97.1%	Clinical and prosthetic stability	5.6% peri-implantitis
Santiago et al., 2025 [49]	Case Series	3	62.3	Maxillary atrophy	N/R	6 months	Custom-designed titanium	High	Stability, patient satisfaction	None observed

**Table 2:** Main characteristics of the complications associated with subperiosteal implants.

Patient	Sex	Age	Early Complications (≤3 months)	Late Complications (>3 months)	Management
1	M	68	Abscess in zone 13 at 3 months	Mucosal dehiscence at 13, 15, 17	Antibiotics, soft tissue monitoring
2	M	27	None	Mucosal dehiscence (lingually)	Improved hygiene
3	M	55	None	Mucosal dehiscence (upper left implant, 2nd abutment)	Improved hygiene
4	F	69	None	None	N/A
5	M	54	None	None	N/A
6	F	60	None	None	N/A
7	F	63	None	None	N/A
8	F	76	None	None	N/A
9	F	64	None	None	N/A

## Data Availability

The original contributions presented in this study are included in the article. Further inquiries can be directed to the corresponding author.
